# Supplementary material for: The influence of age, gender and pharmacogenetic profiles on the perspective on medicines in the German EMPAR study
Source: PLoS One. 2024 Oct 10;19(10):e0311267. doi: 10.1371/journal.pone.0311267 (PMC11466409; doi:10.1371/journal.pone.0311267)
Supplement: S1 Table — Patients who were excluded from the BMQ evaluation either did not fulfil the necessary inclusion criteria, had incomplete BMQ answers which could not be evaluated or IDs could not be identified within the EMPAR population due to terminated participations. (PDF) [file pone.0311267.s001.pdf]

Supplementary Material

The influence of age, gender and pharmacogenetic profiles on the perspective on medicines in the German EMPAR study

Veronica Atemnkeng Ntam[1¶], Tatjana Huebner*[1 ¶], Michael Steffens[1], Christoph Roethlein[1], Britta Haenisch[1,2,4], Julia Stingl[3,4], Roland Linder[5], Catharina Scholl[1].

[1] Research Division, Federal Institute for Drugs and Medical Devices, Bonn, North Rhine-Westphalia, Germany.

[2] German Center for Neurodegenerative Diseases (DZNE), Bonn, North Rhine-Westphalia, Germany

[3] Institute for Clinical Pharmacology, RWTH Aachen University, Aachen, North Rhine-Westphalia, Germany

[4] Center for Translational Medicine, Medical Faculty, University of Bonn, Bonn, North Rhine-Westphalia, Germany

[5] Techniker Krankenkasse (TK), Hamburg, Germany

Table 1. The distribution of male and female participation and dropouts.

	N (%)
Initial questionnaire population	10899
Male	6357 (58.3%)
Female	2966 (27.2%)
Dropouts (Male)	799 (7.3%)
Dropouts (Female)	510 (4.7%)
Dropouts (Missing gender/routine care data)	267 (2.4%)

Patients who were excluded from the BMQ evaluation either did not fulfil the necessary inclusion criteria, had incomplete BMQ answers which could not be evaluated or IDs could not be identified within the EMPAR population due to terminated participations.
