# Supplementary material for: The influence of age, gender and pharmacogenetic profiles on the perspective on medicines in the German EMPAR study
Source: PLoS One. 2024 Oct 10;19(10):e0311267. doi: 10.1371/journal.pone.0311267 (PMC11466409; doi:10.1371/journal.pone.0311267)
Supplement: S2 Table — Correlation coefficients are shown for each analysis. * The correlation is significant at the 0.01 level (2-sided t-test). (PDF) [file pone.0311267.s002.pdf]

Supplementary Material

The influence of age, gender and pharmacogenetic profiles on the perspective on medicines in the German EMPAR study

Veronica Atemnkeng Ntam[1¶], Tatjana Huebner*[1 ¶], Michael Steffens[1], Christoph Roethlein[1], Britta Haenisch[1,2,4], Julia Stingl[3,4], Roland Linder[5], Catharina Scholl[1].

[1] Research Division, Federal Institute for Drugs and Medical Devices, Bonn, North Rhine-Westphalia, Germany.

[2] German Center for Neurodegenerative Diseases (DZNE), Bonn, North Rhine-Westphalia, Germany

[3] Institute for Clinical Pharmacology, RWTH Aachen University, Aachen, North Rhine-Westphalia, Germany

[4] Center for Translational Medicine, Medical Faculty, University of Bonn, Bonn, North Rhine-Westphalia, Germany

[5] Techniker Krankenkasse (TK), Hamburg, Germany

Table 2: Correlation analysis in the BMQ-General 12 and PSM

	Overuse	Harm	Benefit	PSM
Overuse	1	0.47*	-0.13	0.24*
Harm	0.47*	1	-0.23*	0.26*
Benefit	-0.13*	-0.23*	1	-0.11*
PSM	0.24*	0.26*	-0.11*	1

Correlation coefficients are shown for each analysis. * The correlation is significant at the 0.01 level (2-sided t-test)
