# Supplementary material for: The influence of age, gender and pharmacogenetic profiles on the perspective on medicines in the German EMPAR study
Source: PLoS One. 2024 Oct 10;19(10):e0311267. doi: 10.1371/journal.pone.0311267 (PMC11466409; doi:10.1371/journal.pone.0311267)
Supplement: S3 Table — (PDF) [file pone.0311267.s003.pdf]

Supplementary Material

The influence of age, gender and pharmacogenetic profiles on the perspective on medicines in the German EMPAR study

Veronica Atemnkeng Ntam[1¶], Tatjana Huebner*[1¶], Michael Steffens[1], Christoph Roethlein[1], Britta Haenisch[1,2,4], Julia Stingl[3,4], Roland Linder[5], Catharina Scholl[1].

[1] Research Division, Federal Institute for Drugs and Medical Devices, Bonn, North Rhine-Westphalia, Germany.

[2] German Center for Neurodegenerative Diseases (DZNE), Bonn, North Rhine-Westphalia, Germany

[3] Institute for Clinical Pharmacology, RWTH Aachen University, Aachen, North Rhine-Westphalia, Germany

[4] Center for Translational Medicine, Medical Faculty, University of Bonn, Bonn, North Rhine-Westphalia, Germany

[5] Techniker Krankenkasse (TK), Hamburg, Germany

Table 3: Multiple comparisons of the collectives with Tukey's Honest Significant Difference Analysis (HSD)

			Mean difference (I-J)	Std. Error	Sig.	95% confidence interval	
						Lower Limit	Higher Limit
Overuse	Anticoagulant/ antiplatelet drugs	Cholesterol-Lowering Drugs	-0.075	0.070	0.528	-0.24	0.09
		ICD-Y57.9! Diagnosis	-.565*	0.122	**<0.001**	-0.85	-0.28
	Cholesterol-Lowering Drugs	Anticoagulant/ antiplatelet drugs	0.075	0.070	0.528	-0.09	0.24
		ICD-Y57.9! Diagnosis	-0.490*	0.134	**<0.001**	-0.81	-0.18
	ICD-Y57.9! Diagnosis	Anticoagulant/ antiplatelet drugs	0.565*	0.122	**<0.001**	0.28	0.85
		Cholesterol-Lowering Drugs	0.490*	0.134	**<0.001**	0.18	0.81
Harm	Anticoagulant/ antiplatelet drugs	Cholesterol-Lowering Drugs	-0.020	0.067	0.953	-0.18	0.14
		ICD-Y57.9! Diagnosis	-0.247	0.118	0.092	-0.52	0.03
	Cholesterol-Lowering Drugs	Anticoagulant/ antiplatelet drugs	0.020	0.067	0.953	-0.14	0.18
		ICD-Y57.9! Diagnosis	-0.227	0.130	0.186	-0.53	0.08

	ICD-Y57.9! Diagnosis	Anticoagulant/ antiplatelet drugs	0.247	0.118	0.092	-0.03	0.52
		Cholesterol-Lowering Drugs	0.227	0.130	0.186	-0.08	0.53
Benefit	Anticoagulant/antiplatelet drugs	Cholesterol-Lowering Drugs	0.031	0.055	0.834	-0.10	0.16
		ICD-Y57.9! Diagnosis	-0.266*	0.096	**0.015**	-0.49	-0.04
	Cholesterol-Lowering Drugs	Anticoagulant/ antiplatelet drugs	-0.031	0.055	0.834	-0.16	0.10
		ICD-Y57.9! Diagnosis	-0.298*	0.105	**0.013**	-0.54	-0.05
	ICD-Y57.9! Diagnosis	Anticoagulant/ antiplatelet drugs	0.266*	0.096	**0.015**	0.04	0.49
		Cholesterol-Lowering Drugs	0.298*	0.105	**0.013**	0.05	0.54
PSM	Anticoagulant/antiplatelet drugs	Cholesterol-Lowering Drugs	-0.053	0.084	0.807	-0.25	0.15
		ICD-Y57.9! Diagnosis	-0.658*	0.149	**<0.001**	-1.01	-0.31
	Cholesterol-Lowering Drugs	Anticoagulant/ antiplatelet drugs	0.053	0.084	0.807	-0.15	0.25
		ICD-Y57.9! Diagnosis	-0.605*	0.163	**<0.001**	-0.99	-0.22
	ICD-Y57.9! Diagnosis	Anticoagulant/ antiplatelet drugs	0.658*	0.149	**<0.001**	0.31	1.01
		Cholesterol-Lowering Drugs	0.605*	0.163	**<0.001**	0.22	0.99
Natural Remedy	Anticoagulant/antiplatelet drugs	Cholesterol-Lowering Drugs	-0.109	0.057	0.135	-0.24	0.02
		ICD-Y57.9! Diagnosis	-0.211	0.100	0.089	-0.45	0.02
	Cholesterol-Lowering Drugs	Anticoagulant/ antiplatelet drugs	0.109	0.057	0.135	-0.02	0.24
		ICD-Y57.9! Diagnosis	-0.102	0.110	0.623	-0.36	0.16
	ICD-Y57.9! Diagnosis	Anticoagulant/ antiplatelet drugs	0.211	0.100	0.089	-0.02	0.45
		Cholesterol-Lowering Drugs	0.102	0.110	0.623	-0.16	0.36
Gene Test	Anticoagulant/antiplatelet drugs	Cholesterol-Lowering Drugs	0.014	0.024	0.835	-0.04	0.07
		ICD-Y57.9! Diagnosis	-0.048	0.043	0.507	-0.15	0.05
	Cholesterol-Lowering Drugs	Anticoagulant/ antiplatelet drugs	-0.014	0.024	0.835	-0.07	0.04
		ICD-Y57.9! Diagnosis	-0.062	0.047	0.390	-0.17	0.05
	ICD-Y57.9! Diagnosis	Anticoagulant/ antiplatelet drugs	0.048	0.043	0.507	-0.05	0.15
		Cholesterol-Lowering Drugs	0.062	0.047	0.390	-0.05	0.17
