# Supplementary material for: The influence of age, gender and pharmacogenetic profiles on the perspective on medicines in the German EMPAR study
Source: PLoS One. 2024 Oct 10;19(10):e0311267. doi: 10.1371/journal.pone.0311267 (PMC11466409; doi:10.1371/journal.pone.0311267)
Supplement: S4 Table — (PDF) [file pone.0311267.s004.pdf]

Supplementary Material

The influence of age, gender and pharmacogenetic profiles on the perspective on medicines in the German EMPAR study

Veronica Atemnkeng Ntam[1¶], Tatjana Huebner*[1 ¶], Michael Steffens[1], Christoph Roethlein[1], Britta Haenisch[1,2,4], Julia Stingl[3,4], Roland Linder[5], Catharina Scholl[1].

[1] Research Division, Federal Institute for Drugs and Medical Devices, Bonn, North Rhine-Westphalia, Germany.

[2] German Center for Neurodegenerative Diseases (DZNE), Bonn, North Rhine-Westphalia, Germany

[3] Institute for Clinical Pharmacology, RWTH Aachen University, Aachen, North Rhine-Westphalia, Germany

[4] Center for Translational Medicine, Medical Faculty, University of Bonn, Bonn, North Rhine-Westphalia, Germany

[5] Techniker Krankenkasse (TK), Hamburg, Germany

Table 4: Tukey's HSD showing multiple comparisons across the age ranges and the BMQ, PSM, Natural Remedy and Gene Testing scales

Tukey-HSD							
SCALES	**Multiple Age-range comparisions**		**Mean difference**	**Standard erorr**	**Significance**	**95% confidence interval**	
						**Lower limit**	**Upper limit**
Overuse	<36 years	36years - 60years	0.503*	0.177	**0.012**	0.09	0.92
		>60years	0.497*	0.172	**0.011**	0.09	0.90
	36years - 60years	<36 years	-0.503*	0.177	**0.012**	-0.92	-0.09
		>60years	-0.005	0.061	0.996	-0.15	0.14
	>60years	<36 years	-0.497*	0.172	**0.011**	-0.90	-0.09
		36years - 60years	0.005	0.061	0.996	-0.14	0.15
Harm	<36 years	36years - 60years	0.328	0.170	0.131	-0.07	0.73
		>60years	-0.004	0.165	1.000	-0.39	0.38
	36years - 60years	<36 years	-0.328	0.170	0.131	-0.73	0.07
		>60years	-0.332*	0.058	**<0.001**	-0.47	-0.20
	>60years	<36 years	0.004	0.165	1.000	-0.38	0.39
		36years - 60years	0.332*	0.058	**<0.001**	0.20	0.47
Benefit	<36 years	36years - 60years	0.257	0.139	0.153	-0.07	0.58
		>60years	0.206	0.135	0.276	-0.11	0.52
	36years - 60years	<36 years	-0.257	0.139	0.153	-0.58	0.07
		>60years	-0.050	0.047	0.539	-0.16	0.06
	>60years	<36 years	-0.206	0.135	0.276	-0.52	0.11
		36years - 60years	0.050	0.047	0.539	-0.06	0.16
PSM	<36 years	36years - 60years	-0.237	0.214	0.509	-0.74	0.26
		>60years	-0.607*	0.208	**0.010**	-1.10	-0.12
	36years - 60years	<36 years	0.237	0.214	0.509	-0.26	0.74
		>60years	-0.370*	0.073	**<0.001**	-0.54	-0.20
	>60years	<36 years	0.607*	0.208	**0.010**	0.12	1.10
		36years - 60years	0.370*	0.073	**<0.001**	0.20	0.54
Natural Remedy	<36 years	36years - 60years	0.442*	0.144	**0.006**	0.10	0.78
		>60years	0.449*	0.140	**0.004**	0.12	0.78
	36years - 60years	<36 years	-0.442*	0.144	**0.006**	-0.78	-0.10
		>60years	0.007	0.049	0.988	-0.11	0.12
	>60years	<36 years	-0.449*	0.140	**0.004**	-0.78	-0.12
		36years - 60years	-0.007	0.049	0.988	-0.12	0.11

Gene Testing	<36 years	36years - 60years	0.067	0.062	0.527	-0.08	0.21
		>60years	0.152*	0.060	**0.031**	0.01	0.29
	36years - 60years	<36 years	-0.067	0.062	0.527	-0.21	0.08
		>60years	0.085*	0.021	**<0.001**	0.04	0.13
	>60years	<36 years	-0.152*	0.060	**0.031**	-0.29	-0.01
		36years - 60years	-0.085*	0.021	**<0.001**	-0.13	-0.04
