# Supplementary material for: The influence of age, gender and pharmacogenetic profiles on the perspective on medicines in the German EMPAR study
Source: PLoS One. 2024 Oct 10;19(10):e0311267. doi: 10.1371/journal.pone.0311267 (PMC11466409; doi:10.1371/journal.pone.0311267)
Supplement: S5 Table — P-values were calculated within each age group with respect to the collectives. *** = p < 0.001; ** = p < 0.01; * = p < 0.5. (PDF) [file pone.0311267.s005.pdf]

Supplementary Material

The influence of age, gender and pharmacogenetic profiles on the perspective on medicines in the German EMPAR study

Veronica Atemnkeng Ntam[1¶], Tatjana Huebner*[1 ¶], Michael Steffens[1], Christoph Roethlein[1], Britta Haenisch[1,2,4], Julia Stingl[3,4], Roland Linder[5], Catharina Scholl[1].

[1] Research Division, Federal Institute for Drugs and Medical Devices, Bonn, North Rhine-Westphalia, Germany.

[2] German Center for Neurodegenerative Diseases (DZNE), Bonn, North Rhine-Westphalia, Germany

[3] Institute for Clinical Pharmacology, RWTH Aachen University, Aachen, North Rhine-Westphalia, Germany

[4] Center for Translational Medicine, Medical Faculty, University of Bonn, Bonn, North Rhine-Westphalia, Germany

[5] Techniker Krankenkasse (TK), Hamburg, Germany

Table 5: Association of age with patients' perception of medicine within the collectives (2-sided t-test). P-values were calculated within each age group with respect to the collectives

Age Range	N	Overuse Mean (SD)	Harm Mean (SD)	Benefit Mean (SD)	PSM Mean (SD)	Natural Remedy Mean (SD)	Gene Testing Mean (SD)
**<36 years (young adults)**	**227**						
Anticoagulant/antiplatelet	136	12.7 (2.8)	9.2 (2.6)	15.6 (2.0)	9 (3.4)	9.6 (2.4)	4.2 (0.9)
Cholesterol-lowering drugs	17	11.8 (3.2)	8.8 (3.6)	16.4 (2.0)	9.4 (3.4)	8.2 (2.7)	4.2 (0.5)
ICD-10 Y57.9! diagnosis	74	12.6 (3.0)	9.5 (2.5)	15.9 (2.0)	9.5 (3.5)	10.1 (2.3)	4.2 (0.8)
**36-60 years**	**2402**						
Anticoagulant/antiplatelet	1664	12.0 (2.6)*	8.9 (2.5)	15.5 (2.1)	9.3 (3.1)***	9.2 (2.1)	4.2 (0.8)
Cholesterol-lowering drugs	591	12.0 (2.5)	9.1 (2.5)	15.5 (2.0)	9.5 (3.2)***	9.3 (2.0)	4.1 (0.9)
ICD-10 Y57.9! diagnosis	147	12.5 (2.6)	9.2 (2.6)	15.6 (2.0)	10.7 (3.5)	9.3 (2.3)	4.2 (0.8)
**>60 years**	**6694**						
Anticoagulant/antiplatelet	5424	12.0 (2.5)**/***	9.3 (2.4)	15.5 (2.0)*	9.8 (3.0)*	9.2 (2.1)	4.1 (0.9)
Cholesterol-lowering drugs	1032	12.2 (2.5)**	9.3 (2.4)	15.5 (2.0)**	9.8 (3.1)*	9.3 (2.0)	4.1 (0.9)
ICD-10 Y57.9! diagnosis	238	12.7 (2.3)	9.6 (2.4)	15.9 (2.0)	10.4 (3.1)	9.2 (2.1)	4.1 (0.9)

***= p < 0.001; **=p < 0.01; *=p <0.05
