# Supplementary material for: The influence of age, gender and pharmacogenetic profiles on the perspective on medicines in the German EMPAR study
Source: PLoS One. 2024 Oct 10;19(10):e0311267. doi: 10.1371/journal.pone.0311267 (PMC11466409; doi:10.1371/journal.pone.0311267)
Supplement: Attachment — Submitted filename: Response to Reviewers.pdf [file pone.0311267.s007.pdf]

Federal Institute
for Drugs
and Medical Devices

BfArM, Kurt-Georg-Kiesinger-Allee 3, D-53175 Bonn

DIVISION Research
NAME Tatjana, Dr. Hübner
TELEPHONE +49 (0)228 99 307-4327
E-MAIL Tatjana.Huebner@bfarm-research.de

ADDRESS Kurt-Georg-Kiesinger-Allee 3
53175 Bonn
TELEFAX +49 (0)228 99 307-5207
E-MAIL poststelle@bfarm.de
INTERNET www.bfarm.de
Bonn, 30. August 2024

OUR REF 51.1.11

Dear reviewers,
dear editor,

Thank you very much for your comments, suggestions and questions to improve the quality of the manuscript of our research article! Please find our answers and descriptions of implemented changes below.

Journal requirements:

When submitting your revision, we need you to address these additional requirements.
1. Please ensure that your manuscript meets PLOS ONE's style requirements, including those for file naming. The PLOS ONE style templates can be found at https://journals.plos.org/plosone/s/file?id=wjVg/PLOSOne_formatting_sample_main_body.pdf and https://journals.plos.org/plosone/s/file?id=ba62/PLOSOne_formatting_sample_title_authors_affiliations.pdf

Thank you very much, we checked the requirements and adjusted the manuscript accordingly!

2. Thank you for stating the following financial disclosure:
"This EMPAR study was funded by the Innovation Fund of the Federal Joint Committee in Germany (Gemeinsamer Bundesausschuss). Grant number: 01VSF16047."
Please state what role the funders took in the study. If the funders had no role, please state: "The funders had no role in study design, data collection and analysis, decision to publish, or preparation of the manuscript."
If this statement is not correct you must amend it as needed.

Please include this amended Role of Funder statement in your cover letter; we will change the online submission form on your behalf.

We included the suggested statement "The funders had no role in study design, data collection and analysis, decision to publish, or preparation of the manuscript." according to the instructions. Further amendments were not necessary.

3. When completing the data availability statement of the submission form, you indicated that you will make your data available on acceptance. We strongly recommend all authors decide on a data sharing plan before acceptance, as the process can be lengthy and hold up publication timelines. Please note that, though access restrictions are acceptable now, your entire data will need to be made freely accessible if your manuscript is accepted for publication. This policy applies to all data except where public deposition would breach compliance with the protocol approved by your research ethics board. If you are unable to adhere to our open data policy, please kindly revise your statement to explain your reasoning and we will seek the editor's input on an exemption. Please be assured that, once you have provided your new statement, the assessment of your exemption will not hold up the peer review process.

Thank you very much, we prepared the data table S2 Table1 that will be available in the supporting information. We furthermore changed the Email address of the corresponding author, as the old address will soon not be valid.

4. Your ethics statement should only appear in the Methods section of your manuscript. If your ethics statement is written in any section besides the Methods, please move it to the Methods section and delete it from any other section. Please ensure that your ethics statement is included in your manuscript, as the ethics statement entered into the online submission form will not be published alongside your manuscript.

We performed changes to the manuscript according to the instructions.

5. Please include captions for your Supporting Information files at the end of your manuscript, and update any in-text citations to match accordingly. Please see our Supporting Information guidelines for more information: http://journals.plos.org/plosone/s/supporting-information.

We performed changes to the manuscript according to the instructions.

6. Please review your reference list to ensure that it is complete and correct. If you have cited papers that have been retracted, please include the rationale for doing so in the manuscript text, or remove these references and replace them with relevant current references. Any changes to the

reference list should be mentioned in the rebuttal letter that accompanies your revised manuscript. If you need to cite a retracted article, indicate the article's retracted status in the References list and also include a citation and full reference for the retraction notice.
We revised the manuscript according to the instructions.

Reviewers' comments:

Reviewer's Responses to Questions

Reviewer #1: Dear Authors,

Congratulations on your study. some of my comment are as follows:

1. In the title, the word outlook or perspective would better represent views and beliefs.

Thank you very much! We changed the wording accordingly. The new title of the manuscript is "The influence of age, gender and pharmacogenetic profiles on the perspective on medicines in the German EMPAR study"

2. How far back does the retrospective data collected?

Data of a range of 3-4 years was collected for each participant. Data of one year prior to initial prescription of the drugs of interest or the Y57.9! diagnosis, the year of an initial prescription or the Y57.9! diagnosis if it was detected in 2013-2018 and data of one to two subsequent years (for recruited participants in 2018 only data of one subsequent year was available) was provided by the involved health insurance company. Thus, the earliest data was available from 2012 and the latest time at which data was retrieved was 2019. We included the information in the methods section in line 132-135 of the revised version.

3. EMPAR questionnaire: Would be great to have a simple explanation on the questionnaire, such as how many questions for each block. for the gene testing block, is one question enough to understand the participants' prespective?

We adjusted the explanation of the questionnaire in the methods section in line 155-157 of the revised version to improve the information on the number of questions in each block. The gene testing question was not part of a validated questionnaire block such as the Harm, Benefit, Overuse and Perceived Sensitivity to Medicines blocks of the validated BMQ questionnaire. The one question item on gene testing is not sufficient for a validation and thorough assessment of the participants' perspectives on gene testing. We included it to receive an impression of the

participants' attitude towards the use of preemptive testing in routine care. However, for an appropriate evaluation a different study design and an appropriate, validated question block is necessary as here participants explicitly gave their consent to participate in gene testing. These limitations were addressed in the discussion in line 462-471.

4. Figure 3: I find the box plot contains less information than the suppl. table 4. I prefer the table to be in the main manuscript.

Thank you very much! The table is indeed more informative. We removed the box plot and exchanged it with the table. The manuscript and the supplementary material were adjusted accordingly.

5. From the ICD-10 Y57.9! database, does any of the subjects benefited from genotyping? what were their score for gene testing compared to the rest of the population?

The occurrence and frequency of the ICD-10 Y57.9! diagnosis is currently evaluated in terms of a correlation with specific prescriptions and gene variants or phenotypes. The results will be published elsewhere. The score for gene testing in the ICD-10 Y57.9! collective did not differ significantly compared to the other collectives of the EMPAR population.

6. Figure 6 carry very little information on its own. is there correlating data from their ICD code, for example, does the ICD code relate to the variant(s)? does genetic testing solve their ICD code issue?

Figure 6. only shows how the number or accumulation of actionable variants correlates with the evaluated questionnaire scores. Further evaluations in terms of specific ICD 10 codes, medications and relevant actionable variants or phenotypes are currently in progress and will be published elsewhere.

Reviewer #2: This manuscript provides valuable insights into the factors influencing beliefs about medications. All the analyses were well conducted. Following are some minor comments:

1. There should be an explanation of "BMQ" when it is first mentioned in the main context (line 35). I suggest a thorough proofreading. an explanation of "BMQ" when it is first mentioned in the main context (line 35)

We performed a thorough proofreading and checked all abbreviations as suggested. An explanation of "BMQ" was added in line 34 in the revised version when it is first mentioned in the main context. An explanation of "EMPAR" was added in line 27-29, of "ICD" in line 41, of "CYP" in line 193, of "SLCO1B1"in line 195, of "VKORC1" in line 196 of the revised version when

it is first mentioned in the main context. No further unexplained abbreviations at first mentioning were detected.

2. Did the authors considered genetic variations on other genes besides the 4 genes mentioned in the manuscript?

In this manuscript we only focused on the genes and gene variants with the highest evidence (PharmGKB Clinical Annotation Levels of Evidence 1A or 1B) to be actionable with regard to the medication of main study interest (cholesterol lowering drugs and anticoagulants/ antiplatelet agents). Further evaluations including comedications and other relevant genes/gene variants covered by the iPLEX® PGx 74 and the VeriDose® CYP2D6 CNV panel are in progress and will be published elsewhere.

3. There might be additional interesting findings by analyzing a) the association between education levels and patients' beliefs and b) the interaction between different variables.

Thank you very much! The evaluation of the suggested issues is indeed interesting. The patients' beliefs with regard to education levels were assessed; however, as mainly no information on the education level was available and predominantly high education levels were reported in the study population, the results were inconclusive and therefore not included in the manuscript. We included the information on the education level in the discussion section in line 457-460 of the revised version.
The interaction between different variables is currently assessed in terms of specific prescriptions, pharmacogenes and ICD-10 codes and will be published elsewhere.

Thank you for your consideration!

With best regards,

Dr. Tatjana Hübner
